# Legal violence: the struggles of Thai women in Thai massage businesses

**DOI:** 10.3389/fsoc.2024.1208465

**Published:** 2024-02-09

**Authors:** Krittiya Kantachote

**Affiliations:** ^1^Department of Sociology, Srinakharinwirot University, Bangkok, Thailand; ^2^Department of Sociology, University of Southern California, Los Angeles, CA, United States

**Keywords:** legal violence, policy weaponization, intersectionality, government surveillance, Thai massage business, U.S. immigrant communities

## Abstract

Prior studies of legal violence concerning minority groups in the United States are often linked to Immigration Law. Drawing primarily from 30 interviews with Thai massage business owners in Los Angeles, this article reveals how the implementation of local and federal regulations and laws regarding massage businesses constitutes legal violence. Using Crenshaw’s structural, political, and representational intersectionality, this article demonstrates how Thai women’s race, ethnicity, and gender affect their experiences in countering the state regulations. The analysis focuses on two interrelated areas of Thai massage business owners’ and Thai massage therapists’ lives—the strict government surveillance of Thai massage business operations and the enforcement of professional certification. This legal violence toward Thai massage owners and massage therapists is rooted in a legal system that aims to protect consumers. Nevertheless, the effect of legal violence is not limited to undocumented Thai immigrants but also affects Thai American citizens and legal permanent residents.

## 1 Introduction

The term “legal violence” dates back to Cover (1975). Cover (1986) shows that criminal law can damage individuals’ lives through, for example, the subjects’ loss of freedom, property, children, and/or life. Menjívar and Abrego (2012) establish that legal violence is more comprehensive and can go beyond the obvious and direct violent consequences of the law. The term “legal violence” is employed in this paper akin to Menjívar and Abrego (2012), asserting that the convergence of local and federal regulations with criminal law leads to the practice of legal violence. To highlight the nuanced ways through which laws and regulations exert power and control, this manuscript examines the detrimental impacts of laws that can hinder and disrupt immigrants’ pathways to economic mobility and incorporation. The term legal violence is used to describe these consequences because they frequently take detrimental forms that affect immigrants’ livelihood. Further, while there are recorded incidences of physical violence and interpersonal aggression, this article focuses on those that do not result in bodily damage. The analysis focuses on the accumulation of those negative incidents that are not only instantly excruciating but detrimental to immigrants’ long-term chances in the U.S. society. This article traces Thai massage business owners and therapists’ experiences to the execution and the discourses of the laws. To theorize about legal violence, this paper links specific laws and regulations and their implementation to Thai massage business owners and therapists’ work lives.

The notion of legal violence encompasses the diverse and reciprocally strengthening types of violence that the legal system facilitates and intensifies. Through this lens, this article is able to depict the exacerbation of the law’s ordinarily “normal” impact. Furthermore, the legal violence lens highlight the inconsistencies that underly the creation and application of local and federal laws and regulations. The laws and regulations aim to penalize unlicensed massage therapists and massage business owners who violates laws by offering sexual services; yet, unintentionally force them into areas outside the legal system.

Legal violence is both structural and symbolic. Structural in that it is encrypted into the formal legal structures, and symbolic because it is executed by the social order in a way that it becomes normalized, publicly accepted, and respected. Legal violence thus describes the suffering that results from, and is made possible by, implementing laws and regulations that restrict and shape individuals’ daily routines. The existing literature on legal violence concerning minority groups in the United States is often linked to Immigration Law (Takaki, 1990; De Genova, 2004; Miller, 2005; Espiritu, 2008; Menjívar and Abrego, 2012; Cervantes and Menjívar, 2020). Looking beyond Immigration Law, this paper opens up new terrain for studying legal violence within the immigrant community. This article demonstrates how and why federal and state laws lead to injustice and hinder Thai women’s access to economic mobility.

The concept of intersectionality (Crenshaw, 1991) is utilized to understand how legal violence constitutes within the Thai massage community. In many of her works, Crenshaw (1991, 2017) illustrates that experiences and lives cannot be considered separate identities of class, gender, and race; instead, those identities overlap and intersect depending on the context and situation. Consequently, this article considers not only the gender of Thai women, but also their race and ethnicity, and how these affect their experiences when encountering state regulations. This article illustrates how the intersectionality of race, ethnicity, and gender prevents Thai massage owners and therapists^1^ from blending into U.S. society, and to achieve the American dream by operating a legal business. The concept of structural, political, and representational intersectionality is utilized to do so (Crenshaw, 1991, 2017).

Although the laws and regulations regarding massage and spa businesses may not target any particular group, Thai women are greatly affected as they are highly concentrated in this sector. This article shows how government surveillance shapes the operation of Thai massage businesses, in the cause of maintaining community standards, and consumer protection. Legal violence embedded in law enforcement practices hinders Thai massage owners and Thai massage therapists by creating (1) fear and anxiety in operating their businesses, which is harmful to their mental health, (2) conditions that make it nearly impossible to obtain legal professional certification, and (3) constraints to their economic mobility. These difficulties lead Thai women to activate their informal networks, producing instant but temporary solutions. Furthermore, some Thai women are exploited by their coethnics.

As such, the critical scholarly contributions this article makes are twofold. First, the article contributes a new dimension to the scholarship on immigrant ethnic businesses by highlighting the significance of state and federal laws in the type and level of violence against ethnic business operators. Further, the article contributes to applying intersectionality to understand the significance of legal violence in ethnic businesses. On a broad level, this article opens up new avenues for theorizing and critiquing the relationship between state and federal laws, and violence against marginalized groups.

## 2 Literature review

### 2.1 Legal violence in the Thai community

Currently, the literature on the role of the state in regulating immigrant businesses reports three main streams. First, some scholars recognize that the state can be viewed as a resource for ethnic businesses because the state grants visas to immigrants (Zhou, 1992; Seals, 2015). Secondly, the state can be viewed as a source of interethnic and/or racial conflict in ethnic businesses. Many native-born U.S. citizens resent what they see as the government providing certain groups of immigrants a hand up in their quest for mobility with financial resources such as small business loans, tax breaks, and welfare (Portes, 1987; Lee, 2002). Lastly, many scholars examine the role of state surveillance and repression against ethnic businesses. Min (1996), for instance, discusses how the New York State surveilled Korean nail salons in New York when the city began to regulate nail salons in 1991. Estrada and Hondagneu-Sotelo (2011) discuss how street vendors, who are not permitted to operate in Los Angeles, fear police and city authorities. Dhingra (2012) discusses how local government authorities target Indian motel owners because they suspect that the owners allow illegal activities, such as drug dealing and prostitution, on their premises. This article contributes to this literature as it examines how the U.S. government surveils Thai massage owners as well as Thai massage therapists and how that affects business operations. This article discusses how Thai massage owners negotiate and navigate their ways within U.S. laws and regulations.

The constant government surveillance of the Thai community in the United States is not without reason. While the Thai population comprises less than 0.1% of the total U.S. population (U.S. Census Bureau, 2021), the number of lawsuits for human trafficking against Thais is relatively high. For example, between 1998 and 2003, the largest number of foreign victims of trafficking in California came from Thailand (Human Rights Center, 2005). Further, while U.S. human trafficking statistics show that victims came from 47 countries, Thailand is the top source country (Center for Public Policy Studies, 2013).

Another reason for the continual government surveillance of the Thai community could be that massage business is the second largest business sector in which Thai people operate in the United States. According to Bales (2012), massage businesses are one of the many places in Thailand where sex services are available. Kara (2009) claims that Los Angeles has Asian massage businesses that are fronts for prostitution. In addition, he claimed that he had met a sex trafficking victim in a Thai massage business. Between 2012 and 2016, there was a rise in sting operations in massage businesses. The number of Asian-identified people arrested in New York for unlicensed massage and prostitution increased by 2,700% (Dank et al., 2017). Therefore, in the eyes of the U.S. government, Thai massage businesses are perceived as illegal operations, and potential sites for human trafficking.

Under the current regime, inequalities and abuses toward Thai massage owners and therapists are made possible by specific laws and regulations. These structural violence leads to not only instant social suffering but also impede their economic success and integration. Further, these violations are constitutive of symbolic violence. Viewing symbolic violence in accordance with Bourdieu (2001), Thai massage owners and therapists, who are deemed as less dominant in the U.S. society, internalize the existing laws and regulations which leads to social inequality as normal and legitimate. The dominant perpetuates and makes believe that the laws and regulations regarding massage business are legitimate and to be followed. As it is the law, injustices and breaches of rights within the social structure go unchallenged. Thai massage owners and therapists are fully well aware of the power inequalities yet they endure it as the structures are omnipotent and overwhelming. Thai massage owners and therapists learn to accept that their marginalized status is natural and do not try to alter those conditions.

Drawing from structural and symbolic violence concepts, this article contends that legal violence provides the clearest explanation for the experiences of Thai massage owners and therapists. Legal violence captures the suffering on an everyday life caused by and made possible by the corpus of laws and regulations. While the effects of laws and regulations can be considered as both structural and symbolic violence, the author refers to it as legal violence as it is embedded formally and legitimately in legal practices that are perceived as normal and natural. While the laws and regulations aim to maintain community standards and consumer protection, they harm a particular social group—Thai community.

The concept of legal violence is utilized to demonstrate how federal and state laws lead to injustice, and hinder Thai women’s access to economic mobility. Legal violence occurs when laws that aim to control behavior for the general good simultaneously marginalize and harm groups of people. This makes the marginalized groups unprotected, and subject to abuse. Even though the state is not the direct agent of violence, the law permits violence against the targeted group (Abrego and Menjívar, 2011; Menjívar and Abrego, 2012). Legal violence has damaging effects on individual immigrants and their families, both in their everyday lives and in long-term incorporation processes (Menjívar and Abrego, 2012).

Further, intersectionality is applied to understand how and why Thai women are marginalized by their race, ethnicity, and gender. Intersectionality describes the multiple social identities, social forces, and ideological instruments through which power and disadvantage are expressed and legitimized. Intersectionality investigates intersecting power relations of different categories such as age, class, gender, ethnicity, and race as interrelated and mutually influencing one another. Thus, intersectionality is a valuable tool in understanding the complexity of society, and of individuals within a society. The concept allows us to understand social relations across diverse societies as well as individual experiences in everyday life (Crenshaw, 1991; Collins and Bilge, 2020). In this article, the concept of structural, political, and representational intersectionality (Crenshaw, 1991, 2017) is utilized to illustrate how the intersectionality of race, ethnicity, and gender prevents Thai massage owners and therapists from blending into U.S. society, and to achieve the American dream by operating a legal business.

## 3 Data and methods

This research is based primarily on semi-structured interviews with 30 Thai massage business owners in Los Angeles from 2015 to 2017. Interviews and informal conversations with the workers (e.g., managers and therapists) were also used. Research participants were found through phone inquiries and referrals. For diversity, 30 Thai massage business owners were selected from different neighborhoods in Los Angeles County. The interviews included questions on the business owners’ migration histories, motivations for opening an ethnic business, business operations, employment processes, perceptions of gender, race, and ethnicity in the workplace, strategies of market competitions, and plans for the future. The interviews, on average, lasted for an hour. They usually took place at the respondent’s business establishment, in a public setting such as a café or, in a few cases (*N* = 4), by phone. All but one interview was audio-recorded and fully transcribed. The interviews were conducted entirely in Thai, except for one case which was conducted in both Thai and English because the American spouse was present. Atlas.ti software was used to code the interviews and fieldnotes, relying on inductive analysis and grounded theory (Charmaz, 2006). Given the semi-structured interviews, the main themes in this article emerged unprompted.

While most of the respondents were massage business owners and workers, some research participants hold positions of authority in Thai commercial and cultural organizations, which helped understand the community’s perspectives on government surveillance and its effects on Thai society.

The research was supplemented with 5 years of participant observation. Detailed fieldnotes were taken when the author attended Thai business events and gatherings such as the Songkran Festival in Hollywood, and Thai festivals at Thai temples. The author also spent time with Thai people, and had lunch and dinner at local restaurants. In this way, the author observed, interacted, and engaged with people in the Thai community.

### 3.1 Sample characteristics

Table 1 shows the characteristics of sample. Most of the respondents are first-generation immigrants. Seventy-three percent are female, and 27% are male. The respondents’ ages range from 32 to 70, and the average age is 46. Most of them are married with diverse levels of education, ranging from primary education to Ph.D. Forty-seven percent of the respondents have a bachelor’s degree.

**Table 1:** Characteristics of sample.

**Participant**	**Pseudonym**	**Age**	**Sex**	**Marital status**	**Education**	**Visa type to the U.S.**	**Current status**	**Length of stay (years)**	**Business in operation (years)**	**Business type**	**Initial business investment ($)**	**Annual earnings ($)**
1	Bussaba	60s	Female	Divorced	Primary education	Tourist	U.S. citizen	32	16	Family business	20,000	-
2	Dusadee	50s	Female	Married	Bachelor degree	Tourist	Permanent Resident	15	12	Family business	100,000	100,000
3	Kittima	30s	Female	Divorced	Diploma	Student	Permanent Resident	10	3	Family business	21,000	15,000
4	Hatairat	30s	Female	Married	Primary education	Other	Permanent Resident	7	3	Family business	30,000	-
5	Pongsathorn	60s	Male	Divorced	Bachelor degree	Student	U.S. citizen	36	12	Family business	45,000	-
6	Piyanuch	50s	Female	Married	Bachelor degree	Tourist	Permanent Resident	18	7	Family business	15,000	66,000
7	Mingkwan	30s	Female	Married	PhD	Student	U.S. citizen	12	8	Family business	30,000	120,000
8	Korkaew	30s	Female	Single	Bachelor degree	Tourist	Student	9	3	Has shareholder(s)	50,000	60,000
9	Kasem	30s	Male	Married	Bachelor degree	Student	U.S. citizen	10	9	Has shareholder(s)	50,000	65,000
10	Duangdao	40s	Female	Divorced	Master degree	Student	U.S. citizen	10	5	Family business	50,000	30,000
11	Kanokwan	30s	Female	Single	Bachelor degree	Student	U.S. citizen	11	6	Has shareholder(s)	50,000	60,000
12	Duangjai	30s	Female	Divorced	Bachelor degree	Tourist	U.S. citizen	11	11	Family business	25,000	85,000
13	Thongchai	30s	Male	Single	Bachelor degree	Student	Student	10	4	Has shareholder(s)	48,000	144,000
14	Suphanee	60s	Female	Divorced	Junior high	Tourist	U.S. citizen	17	5	Family business	40,000	45,000
15	Suthida	40s	Female	Married	Bachelor degree	Tourist	U.S. citizen	7	3	Family business	60,000	20,000
16	Jaruwan	50s	Female	Married	Diploma	-	U.S. citizen	13	9	Family business	60,000	19,000
17	Boonchai	30s	Male	Married	Master degree	Tourist	Permanent Resident	8	7	Has shareholder(s)	30,000	96,000
18	Premika	30s	Female	Married	Bachelor degree	Student	Permanent Resident	7	3	Has shareholder(s)	30,000	65,000
19	Sureeporn	30s	Female	Married	Master degree	Student	Permanent Resident	11	7	Family business	90,000	100,000
20	Sadudee	60s	Female	Widowed	Primary education	Tourist	U.S. citizen	28	7	Family business	60,000	18,000
21	Chomchanok	50s	Female	Single	Master degree	Tourist	Permanent Resident	10	4	Family business	55,000	-
22	Kornkanok	50s	Female	Divorced	Bachelor degree	Tourist	U.S. citizen	10	9	Family business	30,000	-
23	Anchalee	40s	Female	Married	Junior high	Tourist	U.S. citizen	13	7	Has shareholder(s)	20,000	96,000
24	Panupong	50s	Male	Single	Junior high	Tourist	Permanent Resident	17	6	Family business	50,000	-
25	Pinmanee	30s	Female	Divorced	Bachelor degree	Other	Permanent Resident	9	1	Has shareholder(s)	60,000	54,000
26	Sakorn	50s	Male	Seperated	Senior high	Other	U.S. citizen	24	4	Family business	20,000	-
27	Arisara	40s	Female	Divorced	Bachelor degree	Student	U.S. citizen	20	17	Family business	40,000	60,000
28	Jenjira	40s	Female	Single	Master degree	Student	Permanent Resident	11	5	Family business	30,000	43,800
29	Kaokla	30s	Male	Single	Bachelor degree	Student	U.S. citizen	11	4	Family business	130,000	42,000
30	Jatupong	70s	Male	Married	Diploma	Permanent resident	U.S. citizen	34	8	Family business	45,000	30,000

Pseudonyms and age range are used in this article to preserve participant confidentiality. One person denied telling the visa status when first arrived in the U.S. and seven respondents opted out of telling their annual earnings.

About a third migrated to the United States to further their education, either English language classes or higher education, but most were side-tracked as the work/business opportunity arose. Twenty percent came straight from Thailand for business or work opportunities, hoping to achieve the American dream. Seventeen percent came because they have family members already living in the United States. In terms of travel documents, most Thai business owners first came to the United States on a tourist visa (45%) or a student visa (41%). Fifty-seven percent now have dual citizenship (Thai-American), and 43 % have a green card. Thai business owners have been in the United States for an average of 15 years (median = 11 years), and many speak English fluently.

Thai massage business owners opened their businesses because it requires a relatively small investment compared to other businesses (40%), they have the knowledge and skill in the field (37%), and it yields high profit (27%). On average, the businesses have been in operation for 7 years (median = 6.5 years). While some are relatively new businesses that have been open for only a year, the longest business has been in operation for 17 years.

Most respondents started their businesses using personal savings (80%), while some received loans from their family members, friends, and/or a bank institute. Only one mentioned rotating savings and credit associations (ROSCAs). This is in contrast to Korean and Chinese business owners (Light, 1972; Light and Bonacich, 1991). The initial investment in their massage businesses ranges from $15,000 to $130,000, with the mean being $46,133 (median = $ 45,000). After deducting all related expenses, the business earnings per year range from $15,000 to $144,000. However, the mean is $62,339, and the median is $60,000.

Business scales range from small family-owned businesses that hire no workers to larger establishments with 30 employees. On average, Thai massage business owners hire 10 workers, of which 86% are female. It is common for massage businesses to hire only women, and most (97%) Thai massage businesses hire only Thai staff.

It is important to note that the sample consists of Thai business owners who voluntarily agreed to an interview, so there may be some selection bias. For example, business owners who responded may be law-abiding, while those who break the law by offering sex services, or in some other way, chose not to respond.

## 4 Results

### 4.1 Government surveillance and legal violence toward Thai massage businesses

Before the year 2000, spas and massages were considered services for the upper middle and upper class. Once Thais entered this business niche, massage became an affordable luxury for a wide range of customers from different socioeconomic strata. As a result, these businesses can be found in Beverly Hills and in East and South Los Angeles. However, while Thai massages are well-known among people in Los Angeles, little is known about the struggle that Thai business owners and Thai massage therapists face in business operations, particularly concerning laws and regulations. This section discusses how government surveillance occurs in Thai businesses, and how Thai business owners negotiate and continue their businesses under such strict surveillance. This article describes how government surveillance leads to legal violence toward Thai massage business owners and workers. To contextualize legal violence, this article links specific laws and regulations, and their implementation, to particular outcomes in Thai massage owners’ and massage therapists’ working lives. This article also displays how legal violence comes about in the government’s intention to curb sexual services in the massage industry, and to maintain a massage service standard. Finally, building from Crenshaw’s (1991, 2017) work, this article utilizes the concept of structural, political, and representational intersectionality to understand how and why Thai women are marginalized by their race, ethnicity, and gender.

#### 4.1.1 Thai massage and sex

In the United States, prostitution is illegal except in 10 counties in Nevada (ProCon.org, 2018). However, the media often portrays, and the public often perceives, many legal businesses, such as nightclubs and massage businesses, as places that offer sexual services. Thai massages, in particular, may be depicted as offering sexual services. To prevent sexual solicitation within massage businesses in Los Angeles, the massage ordinance clearly states: “No storage or sale of sexually oriented material and/or sexually-oriented merchandise, as defined by LAMC 103.01, shall be permitted within the Massage Establishment.” (The Los Angeles Police Department, 2015: 8).

Police raids at massage businesses are built into the system and constructed as necessary legal routine practices to keep the community safe from shady businesses. In principal, people and their belongings are shielded from arbitrary government search and seizure under both the United States and California Constitutions. Nevertheless, it is not always required to get a search warrant before conducting a search. Indeed, a majority of police searches are conducted without a warrant. This is allowed in several scenarios, for example, when an officer is clearly witnessing something illegal, when it is part of an arrest, and when an individual grants permission to a search (Los Angeles Police Department, 2022). Thai massage business owners have no choice but to comply to such enforcement as the power dynamics between the police officer and the owner are unequal. Many Thai massage owners comply while being unaware of their legal obligations as they believes noncompliance will put them under the radar of the government. This is when the weaponization of policy occurs.

The weaponization of policy happens when policies harm a specific person or group of people (Faber et al., 2023). This is seen in the case with Thai business owners and Thai massage therapists through several policies (Table 2). Due to ambiguity, weaponized policies can lead to racial discrimination. This is especially so when there are no written rules or the rules are unclear, the people in charge of enforcing them, often those with power, may use different standards which can results in discriminatory outcomes (Okun et al., 2019). The high level of human trafficking and the abuse of coethnics within the Thai community has led the government to construct Thais as legal suspects. This puts Thai massage owners in a difficult position, as officials are prejudiced, and are automatically suspicious of their businesses. The police has the power to conduct unannounced inspections and raids on Thai massage businesses because they suspect that sexual services are provided.

**Table 2:** Policy weaponization.

**Ambiguity discriminatory practices**
– Unannounced inspections and raids on Thai massage businesses.
– Unfair arrests and police brutality.
**Barriers to entry**
– CAMTC certification
1. Attend a minimum of 500 hours at a CAMTC-approved school.
2. Pass a CAMTC-approved exam.

The structural intersectionality of Thai women’s race, ethnicity, and gender make them highly vulnerable to police raids. According to the interviews with Thai massage business owners, many owners and therapists face unfair arrests and police brutality. Police treat the owners and therapists like criminals with no dignity and respect. They are also subject to symbolic violence because they live in fear and believe they have no rights but to accept the abuse as normal. Indeed, the president of the Nuad Thai and Spa Association of America explains the scenario that Thai massage owners face as:

So the policemen treat us (Thai massage owners) with no dignity, as if we are criminals. So yesterday, one (massage owner) came and told me about the police raid. Once I heard it, I cried. I felt so angry for her. They talk to you badly and disrespectfully.

Dusadee, a massage business owner in her 50s, who has been in the United States for 15 years, explains her experience of her business getting raid as:

So I hang up the licenses (the massage therapist licenses) and for all massage therapists I paid the tax correctly…So I have seven workers. The authority listed the employees’ name in a book and asked me to sign. I did not check the details. I just signed. Later, I found out there were 15 names. I was confused. My business is this big how can I hire 15 workers? I think they are just out to get money. I paid $17,500 in fine…My friends advised me not to appeal because they said I might end up paying more if I did.

Dusadee’s case shows that her lack of understanding of the legal procedures led her to sign documents without knowing the consequences. As a result, she was accused of hiring workers illegally. Although Dusadee believes that she did not receive justice, she did not appeal as she is afraid that it might affect her chance of getting U.S. citizenship.

On some occasions, undercover police will pretend to be a customer seeking sexual services. While this is helpful for the police in determining which places provide real massages, and which ones are cover-ups for brothels, the result is that Thai massage business owners experience fear and anxiety in their daily business operations. Racial, ethnic, and gender biases are no longer just an interpersonal problem but an institutional one, as shown by the struggles of Thai businesses vis-á-vis the U.S. government.

In some cases, the massage therapist’s inability to communicate well in English, and her body language may be misinterpreted, leading undercover police to think she agrees to sexual services. The massage therapist will then be arrested and charged with prostitution-related offenses, unlicensed massage if she lacks a certification, and/or issues relating to her immigration status (Chin et al., 2019; Solis, 2021). Some Thai massage therapists are undocumented, and some are on student visas. In these cases, even if they had not engaged in sexual services, they would drop complaints and claims of wrongful arrest against the police, as they feared being deported.

Furthermore, undercover police officers sometimes engage sexually with massage therapists during stings, but they seldom use cameras to record the interactions, which is often downplayed in police reports. After sexual engagement with the women, police officers sometimes verbally humiliate and degrade the therapists, dehumanizing and traumatizing the women who the raid is meant to help (MacMillan and Bhattarai, 2021). Thai women view such acts as an abuse of power by the authorities, and thus become suspicious and distrustful of law enforcement. Many Thai women believe that police violence against them is specifically due to their race, ethnicity, gender, and occupation.

All the respondents are aware of the legal consequences of concealing sex services in their businesses. Therefore, they adhere to a strict code of morals and ethics, abide by the law, and permit no sexual conduct on their premises. Nevertheless, the police inspection causes anxiety for business owners, as they face two main challenges: customers who expect and demand sexual services and massage therapists who provide such services.

The fact that some male customers equate Thai massage businesses with brothels is problematic. Kittima,^2^ a massage business owner in her 30s who has been in the United States for 10 years explains the situation:

Some customers will ask it outright, like do you have a “happy ending?…” Some customers will have ways of finding out. For example, asking do you have “pretty young girls?” When I get that question, I will tell them that I recommend they call elsewhere as I do not have that here…The customer would say, “Why not? What if I pay more? I give good tips.” Why is there a why not? [sounding frustrated].

Thai massage business owners screen customers to weed out those seeking sex services. They do so by observing the customers’ speech and/or how they act. Suthida, a massage business owner in her 40s who has been in the United States for 7 years, explains her screening process in detail:

I screen them in the lobby. So I meet lots of people, so I can tell the way they speak and their behavior. Sometimes they will ask do you have “young girls?” If that’s the case, they are coming for sex because if you want a real massage, you would just ask if we have an opening for one customer…Some men would just stare into my face. You know, men staring at your face and asking do you have “young girls,” or can I check out the massage therapists?… I will ask them where the pains are. They may respond, oh, I have a backache. If they are of large built, I will ask can massage therapist step on their backs? So they will know from the queue I give that it’s real massage…Another thing is the pants. I will tell them they must keep the pants on… If they say, “I feel uncomfortable,” I will say, then we cannot give you the massage.

Nevertheless, not all customers express their desires for sexual services from the beginning. This presents a challenge for the massage owner. Some customers will show their true desire only once they are in the room alone with a massage therapist. Kittima explains:

Some customers I really cannot tell. Like this morning, it just happened. So, this guy dressed up really nicely and looked clean, but once he went in for the massage he tried to touch the massage therapist. She said, no, why are you touching me? No, do not touch me. So, he said, I came here because I wanted to have fun.

Many Thai massage business owners express the difficulty of controlling what goes on behind closed doors. If the massage therapists are honest about their profession, they will inform the owners, who will then go in and tell the customer to get dressed and leave. However, some massage therapists will secretly provide the requested services for extra money without the owners’ consent.

Massage therapists who engage in sex services pose another challenge for massage business owners. According to the owners, it is difficult to prevent massage therapists from providing sex services. Most places have doors or curtains because customers want privacy during their massages. Pongsathorn, a massage business owner in his 60s who has been in the United States for 36 years, shares the unorthodox methods he has used to weed out sexual conduct from his massage business:

I secretly put in a CCTV because I suspect something is going on. So, the lady worked here for just a month, but then the customers queued up to the end of the boulevard [indicating she had way too many regular customers] …Also, her customers would not accept anyone else even though she was busy…So I knew that must be it…I always come and check on my business. I observe those who dress sexily and are constantly changing brand name purses.

In many cases, sexual services in massage businesses may occur but without the consent of the business owners. Unfortunately, the law and regulation seldom allow the business owners to present their sides of the story. Despite knowing that some former therapists engage sexually with their customers, many massage owners do not report them to the police, as they fear their license may be revoked. Instead, they fire those therapists who then move to another massage business, and ruin that business’s reputation. Thai massage business owners have every reason to be afraid. There have been many cases in which massage business owners are charged with promoting prostitution merely because they run a business (Solis, 2021). The owners are in a difficult position because they cannot report the massage therapists who offer sex services to the police. After all, if they did, the police would charge them with violating the customers’ privacy by using hidden cameras. As a result, Thai massage business owners believe they lack government protection.

This legal violence is made possible by existing regulations and enforcement systems. Proponents and supporters of these punitive laws and regulations claim that they protect consumers, and the communities in which the businesses are located. Targeted Thai business owners, however, experience these laws and regulations differently. Thai massage business owners have no choice but to accept and comply with the formal legal structures that give the police the right to pay a visit without prior notice. This instills a constant sense of fear for Thai massage business owners. The police raids and inspections have become normalized as part of daily business operations. Thai massage business owners are no different from Indian motel owners in Dhingra’s (2012) study, who claim to be the target of local governments and cannot control what customers do in their motel rooms, e.g., illegal activities and prostitution.

In addition, many Thai women in the study migrated to the United States to attain the American dream by opening a legal business. However, they believe that the state fails to help them adjust and integrate into society. Law enforcement frames their raids and investigations as combatting sex trafficking (Solis, 2021). Ideally, the police raid is supposed to empower women to work in a safe environment, but Crenshaw (1991) has pointed out that political intersectionality can play out differently for women of color because they are positioned in at least two subordinated groups, and because those groups often pursue conflicting political agendas. Therefore, police raids can have the opposite effect on Thai massage therapists who may be undocumented and/or uncertified. Many Thai massage therapists choose this job as they envision a new life for themselves and their families. When authorities shut down massage businesses, it affects both the business owner and the massage therapists. Thai massage therapists may be even more vulnerable if they lose their jobs, and are forced to work in more risky environments. This is the opposite of the state’s intention. The legal violence lens thus exposes the contradictions on which the formulation and implementation of local, state, and federal law rest. While these laws seek to punish women who offer sexual services, they really just push them to spaces outside the law. Government agencies are thus perceived as a tool of repression against Thai ethnic businesses. This leads to a loss of trust in government agencies, not just in terms of business transactions but also in day-to-day living.

Representational intersectionality is the cultural construct of women of color (Crenshaw, 1991). While the authority’s prosecutions of prostitution claim to protect and empower Thai women, it perpetuates the image of Thailand as a country that traffics women, and of Thai women as exotic commodities that can be purchased. To truly empower Thai women, the government has to ensure that the raids and police investigations have translators that enable the women to understand the accusations and defend themselves, and that they have the right to a fair trial.

#### 4.1.2 Professional certification

Currently, the California Massage Therapy Council (CAMTC) certification is the only credential accepted for massage professionals by the State of California. Although state law does not require CAMTC certification for massage therapists to practice, some cities and counties do require this certification (California Massage Therapy Council, 2018a). In 2015, for example, Los Angeles County replaced its city massage permit with the CAMTC certification (The Los Angeles Police Department, 2015). This new requirement has been extremely challenging for Thai massage business owners and Thai massage therapists.

To obtain this certification, the massage therapist must attend a minimum of 500 hours at a CAMTC-approved school, and pass one of the following exams: (1) Board Certification Exam in Therapeutic Massage and Bodywork (BCETMB) (2) Massage and Bodywork Licensing Exam (MBLEx) (3) National Certification Exam for Therapeutic Massage and Bodywork (NCETMB) and (4) New York State Massage Therapy Examination (California Massage Therapy Council, 2018b).

While obtaining the CAMTC certification seems relatively straightforward, many Thai massage therapists find it arduous. Many Thai massage therapists and massage business owners^3^ admit that they have difficulty obtaining the certificate, as the schools they attended are not approved by CAMTC, or they once were but are no longer. Duangdao, a massage business owner in her 40s who has been in the United States for 10 years, put the issue into perspective:

Therapists must graduate from a school approved by the state…that mean you need to take classes with foreign teachers (non-Thai). Most massage therapists who came here and have worked here for a long time and got the city license are the pioneers and do not have much English skills. It is difficult for them to understand the classes and even harder for them to take and pass the written exam. As a result, many massage therapists have given up their hope.

While Thai massage therapists have a choice of four different CAMTC-approved exams, most take the MBLEx exam. According to many massage business owners and therapists, this exam is quite difficult as they must study anatomy and other technical subjects.

The structural intersectionality of Thai women’s ethnicity, race, and class places them at a disadvantage. Despite having worked in this occupation since they arrived in the United States a decade back, many Thai women are now forced to comply with the new legal certification requirements. Thai massage therapists’ educational background is varied. Some therapists have only a few years of education, while others have a college degree or post-graduate qualification. Therapists who have only a few years of education and/or do not understand English well, find it extremely difficult to understand the class materials, pass the exam, and obtain the certificate. This is especially so for older Thai women whose English is not good. As a result, many women are exploited by their coethnics, who sell them fraudulent certificates for several thousand dollars, and only later do they realize that they are useless. This requirement opens opportunities for coethnic exploitation, and can result in the development of toxic immigrant communities (Del Real, 2019; Cervantes and Menjívar, 2020).

The new legislation aims to ensure that massage therapists have the required knowledge to provide safe services to customers. Although the new legislation may not target a specific race, ethnicity, or gender, Thai women are seriously affected because they are highly concentrated in the massage business. To get the CAMTC certification, one must have the money, the time, and the skill to pass the examination in English. Thai massage therapists who are responsible for sending remittances home find it extremely difficult, as the time and money they spend enrolling in a school mean less time to work and earn money.

The current situation that Thai massage therapists in Los Angeles face are similar to what Korean nail salon workers in New York experienced three decades ago. In 1991, the New York State Legislature started to regulate nail salons. The law required six hundred hours of education and a qualification examination to earn the nail specialty license. Nonetheless, the Korean Nail Salon Association of New York successfully lobbied for a grandfather clause. The clause allowed nail salon workers who could prove work experience of one or more years in nail salons to obtain licenses without additional classes and/or examinations (Min, 1996; Kang, 2010). Unfortunately, Thai massage therapists are not as lucky. Despite having the Nuad Thai and Spa Association of America to advocate for their rights, the association has been less successful in getting a similar clause to exempt Thai therapists with prior work experience.

The CAMTC certification requirement of massage therapists shows that the weaponization of policy occurs not only when the rules are arbitrary but even so when there are clear written rules. These written rules, nevertheless, can be discriminatory. Even with fair application of the regulations, there will always be an overt discriminatory consequence. Standardized tests have proven to be the most successful technique since they seem fair on the surface. If someone is deemed to be “unqualified,” it is reasonable to deny them the job opportunities (Faber et al., 2023). As the CAMTC classes and exams are conducted in English, this puts Thai massage therapists at a great disadvantage. This case is similar to the requirement for psychologists in Canada to pass a French language exam in order to work. However, the ability to be a good psychologist has nothing to do with the language ability (Olson, 2020; Faber et al., 2023). Likewise, the ability to be a good massage therapist has nothing to do with the language ability. In addition, while fining unlicensed massage therapists on the surface may seems fair yet because Thai massage places are inspected more so than other massage places, the chances of Thai massage therapists getting fine is higher.

Law enforcement has had detrimental effects not only on Thai massage therapists, but also on Thai massage business owners. While the certification scheme aims to punish uncertified massage therapists, it leads to legal violence toward the therapists and the business owners. Moreover, since the requirement was enforced, Thai massage business owners have encountered difficulties finding certified massage therapists. Duangdao elucidates:

So those who got the permit from the city and used to be able to work now cannot because they do not have the state certification. The state certification is really difficult to get. The problem now is that we lack massage therapists, which forces many massage businesses to close and sell off because we could not find massage therapists with state certification. Massage therapists also have problems because they cannot work, so they sneak and do it. But once the police raid, they get a ticket and must pay a fine. So if they get fined a couple of times, it affects their chances of getting the state certification.

While understanding the reasons for the policy change, many Thai massage business owners believe they are not given much choice but to hire uncertified therapists. Based on my sample, only one owner got fined for hiring uncertified therapists, but many Thai massage business owners are worried that they might be the next in line to get into trouble with the state. Thai massage business owners believe that, if they get into trouble with the authorities, their sentence would be more severe than that of a white person due to their lack of understanding of the U.S. legal system and their inability to defend themselves in court. Therefore, to be on good terms with the government and give themselves a sense of security, most Thai massage business owners strategize by hiring both certified and uncertified massage therapists.

Most Thai massage business owners support their uncertified therapists in getting certified. Suphanee, who is in her 60s, and has been in the United States for 17 years, explains:

I support uncertified massage therapists to take that three-month course because you have to do this all your lives. So, the law already came out, and if you do not have the certificate, massage business owners will not dare hire you in the future. So, I recommend that they sacrifice some working hours and take the courses two to three days per week. Also, now we have the spa group, which helps Thai people. So there will be a Thai version of the MBLEx exam.

This massage business owner is quite optimistic about a Thai version of the MBLEx exam. However, the exam is still available only in English and Spanish (Federation of State Massage Therapy Boards, 2022).

Crenshaw (1991) points out that political and representational intersectionality can come at a cost to women of color. Women of color have trouble getting their voices heard, and their experiences incorporated into agendas. Sometimes what society claims to be best for women of color is in reality an obstacle to their progress. The authorities claim that the certification scheme will put certified massage therapists at an advantage, and uplift the massage therapists’ image and status in society, but this has had a detrimental effect on Thai women. The professional certification does not only reduce competition, but eliminates the only source of income for some Thai women, as many massage business owners will hire therapists with a certificate if given a choice. As a result, many Thai women have lost their jobs, and some who still insist on working in this niche have to hide like criminals. While legal violence is rooted in the legal system that purports to protect consumers, it produces unintended economic and emotional injury to the Thai community. To save the Thai massage community, the government could exempt therapists with prior work experience from the MBLEx exam, or conduct the exam in Thai.

Using the legal violence lens, this article unearths the deleterious consequences of a system of laws and policies on the massage profession certification scheme. While the certification is enforced to protect consumers, this regulation threatens the livelihood of Thai massage therapists and Thai small business owners. Because obtaining the certificate is difficult, some therapists have decided to remain uncertified. This is especially the case for the pioneers who have worked for decades as massage therapists, and were looking forward to their retirement within the next few years. By being uncertified, many Thai women face downward mobility as Thai massage business owners prefer hiring certified massage therapists to avoid getting fined and blacklisted by the government.

Thai massage business owners also risk downward mobility as many have given up hope, and plan to sell off their businesses due to the lack of certified massage therapists. When asked what they will do once they sell off their businesses, some said they would go back and work as massage therapists because that is the only skill they have. Therefore, this new regulation of professional certification leads to legal violence, which prevents Thai massage therapists from earning a living using their existing skills, and prevents them from becoming future business owners. This regulation thus hinders the economic mobility of Thai women. The effect of legal violence is not limited to undocumented Thai immigrants, but also Thai-American citizens and Thai legal permanent residents in the massage sector. These effects are harmful to the Thai community in the short and long term.

## 5 Discussion and conclusion

In this article, the concept of legal violence and intersectionality is utilized to understand how laws and regulations regarding the massage business affect the Thai community in Los Angeles, California. Viewing Thai massage owners’ and therapists’ race, ethnicity, and gender not as distinct characteristics, but as intersecting and interrelated elements that continue to influence one another allows for a nuanced understanding of the complexity of interrelated power influencing the Thai massage community (Crenshaw, 1991, 2017; Collins and Bilge, 2020). Previous research on legal violence against minority groups in the United States is often linked to how immigration law is used as a tool by the state toward undocumented immigrants (De Genova, 2004; Miller, 2005; Menjívar and Abrego, 2012; Cervantes and Menjívar, 2020). This article shows that the concept of legal violence is much more comprehensive, and can be applied to a broader range of situations. The concept is utilized to explain how the implementation of local and federal regulations and laws constitutes legal violence. This study advances the concept of legal violence by showing that legal violence affects not only undocumented immigrants, but also legal residents—Thai U.S. citizens as well as Thai permanent residents—in the massage business sector. This opens up the platform for global scholars of all fields to utilize and develop this concept to fully understand the existing and evolving social phenomenon. Further, Crenshaw’s (1991) concepts of structural, political, and representational intersectionality are utilized to understand how and why Thai women’s race, ethnicity, and gender affect their experience in countering state regulations. The implementation of government surveillance on Thai massage businesses and the implementation of certification for the massage profession is intended to protect customers and society from unlawful sexual transactions, and maintain a standard of massage services. These laws and regulations are well structured, and have become normalized and publicly accepted. However, these laws and regulations severely affect Thai massage business owners and therapists. They lead to constant fear, anxiety, distrust of government authority, and constrain the economic mobility of Thai women. Thus, legal violence has damaging effects on Thai massage business owners, therapists, and their families, both in their everyday lives and in their long-term prospects.

This article has two policy recommendations that can benefit the Thai community and the U.S. government (see Table 3). First, it concerns sexual services in Thai massage places. This is one of the biggest challenges for Thai massage business owners as sometimes massage therapists provide sexual services without the owners’ consent. Most Thai massage business owners claim they do their best to prevent sexual transactions by screening both customers and therapists. Nevertheless, when the police raid the business and find that sexual services are offered, both the owners and massage therapists are prosecuted. Here, the state can provide a contact channel for massage business owners to inform the state of suspected sexual conduct and give a fair investigation without immediate charge. By doing so, massage business owners will trust the legal authority and cooperate to promote sex-free massage places in the community.

**Table 3:** Policy recommendations.

**System reform**
– Provide a translator during a raid.
– Provide a contact channel for massage business owners to inform the state of suspected sexual conduct and give a fair investigation without immediate charge.
**Reduce barriers to entry**
– Reconsider the need for the CMATC-approved exams for Thai therapists with prior work experience to reduce financial and language burden.
– Encourage CAMTC-approved schools to conduct classes in Thai language.
– Introduce the MBLEx exam in Thai language.

Second, as massage and spa businesses are the Thai community’s second-largest business, there should be a policy to help Thai massage therapists get certified. One of the criteria to obtain the CAMTC certification is to pass one of the CAMTC-approved exams. Most Thai massage therapists take the MBLEx exam. However, the MBLEx exam is still available only in English and Spanish (Federation of State Massage Therapy Boards, 2022), which leads to unequal opportunities for Thai, especially older Thai women who may not have English proficiency. The exam aims to test whether massage therapists have knowledge and skills regarding their occupation, not test their English proficiency. Thus, the exams should be available in Thai as massage is an occupational niche Thai specializes in. Having the exam in the Thai language will be beneficial to the Thai community in helping Thai massage therapists get certified and to the consumers who can be confident that they receive the service from a person with mandatory knowledge. Future research could examine whether other ethnic groups in the massage business sector in Los Angeles also face similar barriers. Further, comparative studies can be conducted to see whether Thai massage business owners and therapists in other states face similar obstacles.

## Positionality statement

The author is a Thai sociologist who does research on Thai community in Los Angeles, California. Being Thai facilitated her rapport with the respondents. Her shared racial and ethnic status helped her respondents to feel comfortable so they openly discussed their business situations, and the problems they faced with government authorities in operating their businesses.

## Data availability statement

The datasets analyzed in this study are not readily available as they contain participants’ personal information. Requests to access these datasets should be directed to krittiyak@g.swu.ac.th.

## Ethics statement

The studies involving humans were approved by University of Southern California Institutional Review Board. The studies were conducted in accordance with the local legislation and institutional requirements. The participants provided their written informed consent to participate in this study.

## Author contributions

The author confirms being the sole contributor of this work and has approved it for publication.

## Footnotes

^1^The word “massage therapist” is intentionally used instead of “masseuse” or “masseur” as it is gender-neutral. While all the terms refer to a person who provides massage professionally, they have different connotations. The latter two are often linked to sexual services.

^2^Pseudonyms are used in this article to preserve participant confidentiality.

^3^Some Thai massage business owners also work in their businesses as massage therapists.
